# Plasma Cell Mucositis of Oro- and Hypopharynx: A Case Report

**DOI:** 10.1155/2012/304136

**Published:** 2012-06-17

**Authors:** Mark Puvanendran, Anja Lieder, Wolfgang Issing

**Affiliations:** ^1^Department of Otolaryngology, Freeman Hospital, Newcastle NE7 7DN, UK; ^2^Freeman Hospital, Freeman Road, Newcastle NE7 7DN, UK

## Abstract

*Objective*. To raise awareness of plasma cell mucositis as a rare differential diagnosis for oral mucosal ulceration and its macroscopic similarity to malignancy. *Method*. We report a patient who presented with oral features suggestive of malignancy. A biopsy revealed plasma cell mucositis. *Results*. The patient successfully had a full excision of one lesion and a spontaneous resolution of the other. *Conclusion*. With the increasing incidence of oral mucosal pathology, physicians should be aware of this differential diagnosis.

## 1. Introduction

Oral cavity mucosal ulceration is a common condition for which the mainstay of treatment is conservative. Mucositis is painful inflammation and ulceration of the mucous membranes of the upper aerodigestive tract and the gastrointestinal tract, and it is a well-known and recognized complication of radiotherapy and chemotherapy. Oral mucositis is particularly prevalent in head and neck malignancy patients, affecting up to 80% receiving chemo- and radiotherapy [1].

Plasma cell mucositis is a rare variation of mucositis comprising of a polyclonal plasma cell infiltration of the mucosa [2]. The mucosa is intensely erythematous with accompanying papillomatous or nodular surface changes [3]. Histopathologically, there are distinct changes; however, the diagnosis is made from a combination of clinical and pathological findings such as erythematous surface plaques, epithelial hyperplasia, and dense plasmacytic infiltration in the superficial lamina propria [4].

We present a case of plasma cell mucositis occurring during treatment for reflux. The patient presented with two lesions ulceration based around the uvula and a hypopharyngeal lesion. Clinically, it appeared as a squamous cell carcinoma, and an urgent laryngopharyngooesophagoscopy was arranged. The uvula lesion was completely excised, and the hypopharyngeal lesion was biopsied. A review of the literature has been carried out. 

## 2. Case Report

**Figure 1:**
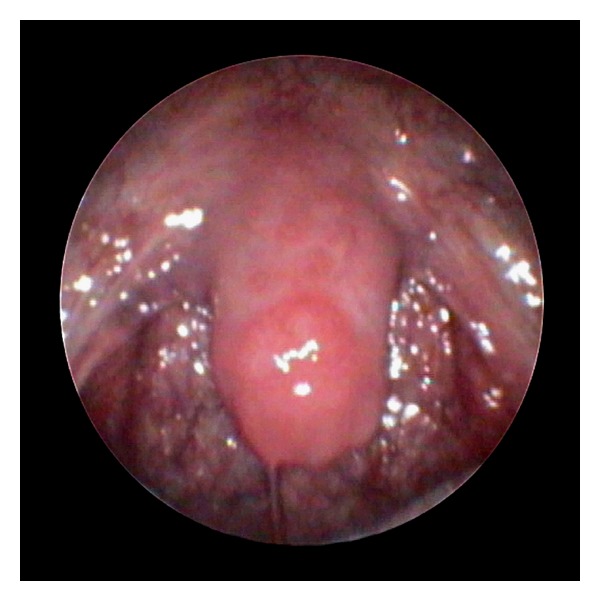
Plasma cell mucositis of the pharynx and uvula (endoscopic photograph).

A healthy 74-year-old man was initially seen in clinic presenting with globus-type symptoms. A detailed history was taken and thorough examination including oral cavity and upper aerodigestive tract performed. He was diagnosed with laryngopharyngeal reflux and commenced on Omeprazole 10 mg twice daily and Gaviscon Advance 10 ml at bedtime. At a review four months later, his globus type symptoms had significantly improved but he was noted to have ulceration of his uvula that raised the clinical suspicion of squamous cell carcinoma (Figure 1). A similar lesion was seen in his right hypopharynx. Urgent laryngopharyngoscopy was arranged and excision biopsies performed. 

The specimens showed hyperplastic squamous epithelium with sheets of plasma cells between the rete processes. Immunohistochemical analysis demonstrated strong expression of CD79a and occasional CD20 expression in plasma cells. The plasma cells were determined to be polyclonal by *insitu* hybridization. The features were in keeping with plasma cell mucositis.

The mucositis involved the uvula and the hypopharyngeal wall. The lesion based at the uvula was completely resected during the biopsy. The lesion of the hypopharyngeal wall was biopsied, and remaining area was managed in an expectant manner. After six months, the uvula showed some slight residual erythema and the hypopharyngeal lesion resolved completely. 

## 3. Discussion

Plasma cell mucositis is an extremely rare condition, with less than 50 cases reported. It is a form of oral mucositis, a painful inflammation and ulceration of the oral cavity, tending to affect adults with an average age of 55 years [5].

It consists of a packed plasma cell infiltrate of the mucous membrane of the upper aerodigestive tract, and similar conditions may involve other membranes, for example, the glans penis. The condition was originally described by Zoon in 1952 [6], and amendments were made by Schuermann and Luders [7, 8]. White et al. further refined the definition to encompass all plasma-cell infiltrates of the mucus membranes of body orifices [9]. 

It is a benign condition, but complications of critical stenosis secondary to plasma cell mucositis, involving the trachea and bronchi, have been reported [10].

Currently, no aetiological causes are known, and a number of theories have been discussed in the literature from a reaction to chewing gum or other foreign substances though allergy testing has proven to be inconclusive [11]. Periodontitis has also been proposed. Patients tend to have a history of autoimmune or immunological dysfunction such as diabetes or seronegative rheumatoid arthritis.

Patients classically present with dysphagia, oral pain, pharyngitis, and persistent hoarseness. The World Health Organization (WHO) oral toxicity score or the National Cancer Institute Common Terminology Criteria for Adverse Events (NCI-CTCAE) for oral mucositis are commonly used to assess the extent of severity [12, 13]. The WHO score grades the severity from 0 (no oral mucositis) to 4 (swallowing not possible, additional nutrition required). Alternatively, the oral mucositis assessment scale (OMAS) may be used, and it is highly reproducible and is responsive over time and accurate in recording associated symptoms [14]. The assessment is based on the appearance and extent of redness and ulceration of the oral cavity.

The mucosa is erythematous with accompanying surface papillomatous cobblestone, nodular or velvet changes. Microscopically, the cells have an acanthotic epidermis and rete ridges. There is a dense submucosal infiltration of mainly mature plasma cells. Russell bodies (immunoglobulin within cytoplasm of plasma cells) have been noted. The benign nature of the condition has been illustrated by immunoperoxidase staining and gene rearrangement studies demonstrating a mixed population of kappa and lambda light chains and various heavy chains.

When considering plasma cell mucositis as a diagnosis, it is important to ensure that other conditions that present in a similar manner are excluded. Alternative conditions include allergic contact mucositis, plasmocitoma, plasmacanthoma and cheilitis granulomatosa. Syphilis and candidiasis are two of the more well-known differential diagnoses. Erythroplasia of Queyrat is usually a form of intraepithelial carcinoma affecting the glans penis; however, cases have been noted in the oral cavity and need to be considered. 

A review of the literature has shown that plasma cell mucositis has been treated with topical [15], intralesional [9, 16], and systemic corticosteroids [3]. Antibiotics [17], antifungals [9, 18], cryotherapy (liquid nitrogen) laser, and excision have also been used. The most frequent treatment modality is corticosteroids, but this has unreliable results and is of questionable benefit. Topical tacrolimus at a concentration of 0.03% has been reported to treat a plasma cell infiltration of the lower lip [19].

## 4. Conclusion

Plasma cell mucositis is a rare differential diagnosis that needs to be considered when treating mucositis that appears refractory to conventional treatment. 

## References

[1] Sonis S, Elting L, Keefe D Burden of illness and economic impact of mucosal injury (MUI) in solid tumour—a multinational prospective observational study design. *Support Care Cancer*.

[2] Bharti R, Smith DR (2003). Mucous membrane plasmacytosis: a case report and review of the literature. *Dermatology Online Journal*.

[3] Ferreiro JA, Egorshin EV, Olsen KD, Banks PM, Weiland LH (1994). Mucous membrane plasmacytosis of the upper aerodigestive tract: a clinicopathologic study. *American Journal of Surgical Pathology*.

[4] Smith ME, Crighton AJ, Chisholm DM, Mountain RE (1999). Plasma cell mucositis: a review and case report. *Journal of Oral Pathology and Medicine*.

[5] Solomon LW, Wein RO, Rosenwald I, Laver N (2008). Plasma cell mucositis of the oral cavity: report of a case and review of the literature. *Oral Surgery, Oral Medicine, Oral Pathology, Oral Radiology and Endodontology*.

[6] Zoon JJ (1952). Chronic benign circumscript plasmocytic balanoposthitis. *Dermatologica*.

[7] Schuermann H (1960). Plasmacytosis circumorificialis. *Dtsch Zahnarztl*.

[8] Luders G (1972). Plasmocytosis mucosae: ein oft verkanntes neues Krankheitsbild. *Munch Med Wochenschr*.

[9] White JW, Olsen KD, Banks PM (1986). Plasma cell orificial mucositis: report of a case and review of the literature. *Archives of Dermatology*.

[10] Lucarelli MR, Allen JN, Magro CM (2005). Plasma cell mucositis of the distal airways. *Thorax*.

[11] Sollecito TP, Greenberg MS (1992). Plasma cell gingivitis: report of two cases. *Oral Surgery Oral Medicine and Oral Pathology*.

[12] World Health Organization (1979). *Handbook for Reporting Results of Cancer Treatment*.

[13] National Cancer Institute ( National Institutes of Health) US National Cancer Institute Common Toxicity Criteria. http://ctep.cancer.gov/.

[14] Sonis ST, Eilers JP, Epstein JB (1999). Validation of a new scoring system for the assessment of clinical trial research of oral mucositis induced by radiation or chemotherapy. *Cancer*.

[15] Jones SK, Kennedy CT (1988). Response of plasma cell orificial mucositis to topically applied steroids. *Archives of Dermatology*.

[16] Kaur C, Thami GP, Sarkar R, Kanwar AJ (2001). Plasma cell mucositis. *Journal of the European Academy of Dermatology and Venereology*.

[17] Mahler V, Hornstein OP, Kiesewetter F (1996). Plasma cell gingivitis: treatment with 2% fusidic acid. *Journal of the American Academy of Dermatology*.

[18] Palmer RM, Eveson JW (1981). Plasmacell gingivitis. *Oral Surgery Oral Medicine and Oral Pathology*.

[19] Jin SP, Cho KH, Huh CH (2010). Plasma cell cheilitis, successfully treated with topical 0.03% tacrolimus ointment. *Journal of Dermatological Treatment*.

